# Determination of the Sensory Characteristics of Traditional and Novel Fortified Blended Foods Used in Supplementary Feeding Programs

**DOI:** 10.3390/foods8070261

**Published:** 2019-07-17

**Authors:** Sirichat Chanadang, Edgar Chambers IV

**Affiliations:** 1 Faculty of Agricultural Product Innovation and Technology, Srinakharinwirot University, Bangkok 10110, Thailand; 2 Department of Food, Nutrition, Dietetics and Health, College of Human Ecology, Kansas State University, Manhattan, KS 66506, USA

**Keywords:** fortified blended foods (FBFs), sensory, food aid, extrusion, cereal, legume, infant, child, porridge

## Abstract

Despite the wide use of traditional non-extruded fortified blended foods (FBFs), such as corn soy blend plus (CSB+), in supplementary feeding programs, there is limited evidence of its effectiveness on improving nutritional outcomes and little information on actual sensory properties. Fifteen novel extruded FBFs were developed with variations in processing and ingredients in order to improve the quality of food aid products based on the Food Aid Quality Review (FAQR) recommendations. Descriptive sensory analysis was performed to determine the effects of the processing parameters and ingredients on the sensory properties of traditional and novel FBFs. The extrusion process affected the aroma and flavor of the tested products. Novel FBFs from the extrusion process had more pronounced toasted characteristics, probably because of the high temperature used during extrusion. The ingredient composition of the FBFs also had a significant impact on the sensory properties of the products. The addition of sugar to novel FBFs leads to a significant increase in sweetness, which could improve acceptance. The level of lipids in binary blends appeared to be mainly responsible for the bitterness of the product. In addition, legumes, which were a primary ingredient, contributed to the beany characteristics of the products. The higher amounts of legume used in the formulations led to beany characteristics that could be perceived from the products and could be a negative trait depending on consumers’ prior use of legume-based products.

## 1. Introduction

Food insecurity around the world is always increasing due to many causes, including growing populations, poverty, and natural disasters [1]. The State of Food Insecurity in the World, 2015 reported that approximately 795 million people in the world were undernourished in 2014–2016 [2]. Fortified blended foods (FBFs) were developed in the 1960s by the United States Agency for International Development (USAID, Washington, DC, USA) to provide a supplement for young children who suffered from moderate acute malnutrition in many developing countries around the world [3,4]. The most commonly distributed cereal based FBF by USAID is a corn-soy blend (CSB) which consists of corn (75–80%) as a source of carbohydrate and soy (20–25%) as a source of protein. Although FBFs form an important part of the food aid ration, there is limited evidence of their abilities in treating young children with malnutrition [3,4,5] and little information on their sensory properties. 

The Food Aid Quality Review (FAQR) in 2011 by Webb et al. [6] recommended changing the formulation of existing FBFs in order to improve their nutritional quality. These recommendations included adding animal-source protein to promote linear growth of children, increasing fat content through the addition of vegetable oil, adding a flavor enhancer to formulations to improve the acceptability of FBFs, and upgrading micronutrient compositions in FBFs. In addition, the decortication of cereals and legumes used in FBFs is recommended in order to reduce the fiber content and eliminate phenolic compounds that can reduce the energy intake and lower protein digestibility and mineral absorption [5].

Another recommendation from Webb et al. [6] was to increase the solids content of FBFs to 20% to increase the nutrient content. However, porridge prepared from the current FBFs at this concentration is too viscous for consumption by infants and young children [7]. Mothers normally add more water into porridge to make it more drinkable before feeding it to their children, which results in a low nutritional value and energy density [5]. Extrusion cooking of starchy ingredients for FBFs can result in less viscous cooked porridge, making them more ideal for delivering higher density energy meals at lower viscosities for infants and young children [8]. Extruded products also require short cooking times and less fuel [5], which makes them more valuable to people with limited time and energy sources.

Webb et al. [6] also encouraged the exploration of new grains or legumes that could be used beyond the traditional FBFs, including CSBs and wheat-soy blends (WSB). Corn has been used as the main staple for current FBFs because it is a good source of starch, plant-based protein, dietary fiber, B vitamins, and is available in bulk for the food aid program [9,10]. However, the high demand of corn for many uses, especially for fuel production, makes the prices increase [11] and this directly affects food aid commodities. Heat-treated soy in full fat form or defatted flour is primarily used as a source of protein in FBFs. However, soy may contain high levels of anti-nutritional factors, such as phytate and phytoestrogen, which have unknown long-tern health effects [9]. 

Sorghum has been examined as a potential alternative ingredient in FBFs with a number of advantages over corn, including higher levels of protein, fat, and some micronutrients when processed properly [12,13]. Cowpea has also been considered as an alternative legume that can be used in FBFs because of the high levels of protein, energy, and other nutrients [14]. Sorghum and cowpea are cultivated and consumed as part of human foods in many parts of developing countries [14,15]. Therefore, populations in these areas should be familiar with the flavor of sorghum and cowpea, which makes these good candidates for use in FBFs. Moreover, both sorghum and cowpea are mostly non-genetically modified organism (GMO) crops, which allows them to be used in many countries that have banned the use of GMO products.

Recent work has shown that various types of extruded FBFs made with sorghum or corn and cowpea or soy are at least as preferred as CSB+ by children in Tanzania [16]. In addition, data has shown that the shelf-life of such products is generally 24 months or greater [17], far exceeding the required shelf-life for such products.

Based on the recommendations of the FAQR, fifteen newly formulated, extruded FBFs with varied processing techniques and ingredients were developed. The objective of this study was to determine the effects of processing techniques (extrusion vs. non-extrusion, milling type, decortication process, and the step of adding antioxidant to the product) and ingredients on the sensory properties of traditional and novel FBFs.

## 2. Materials and Methods 

### 2.1. Samples

Fifteen novel extruded FBFs and one current-non extruded FBF were used in this study. These products were potential variations in a large feeding trial in Tanzania to test sorghum cowpea blends against other products [18].

#### 2.1.1. Novel Extruded Fortified Blended Foods

Fifteen possible extruded FBFs varied in milling type, decortication process, the order of adding antioxidant to the blends, and ingredients are shown in Table 1.

The whole grains—sorghum varieties V1 (Fontanelle 4525), V2 (738Y), V3 (217X Burgundy) (Nu Life Market, Scott City, KS, USA), and corn (Agronomy Foundation Seed, Kansas State University, Manhattan, KS, USA) were used for pilot milling at Hall Ross Flour Mill (Kansas State University, Manhattan, KS, USA) to obtain whole and decorticated flours. Soybeans (Kansas River Valley Experiment Field, Kansas State University, Manhattan, KS, USA) and cowpea grains (LPD Enterprises LLC, Olathe, KS, USA) were milled at Hall Ross Flour Mill (Kansas State University, Manhattan, KS, USA). Commercially milled whole and decorticated sorghum flour variety V1 were obtained from Nu Life Market, Scott City, KS, USA. Commercially milled degermed corn flour and whole corn flour were purchased from Agricor, Marion, Indiana, USA. Defatted soy flour was purchased from American Natural Soy, Cherokee, IA, USA. 

The cereal/legume flours were blended. For seven sorghum-cowpea blends, one of the three sorghum varieties of flour, whole or decorticated, was mixed with cowpea flour. For five sorghum-soy blends, sorghum variety V1, whole or decorticated, was mixed with low fat (1.85%), medium fat (6.94%), or full fat (16.93%) soybean flour. For three corn-soy blends, whole or degermed corn flour with medium fat and full fat soybean flour were used. All binary blends were extruded on a single screw extruder X-20 (Wenger Manufacturing Inc., Sabetha, KS, USA) at a screw speed ranging from 500–550 rpm with 18–24% process moisture. The extrudates were cut at the die exit with a face-mounted five blade rotary knife, and dried in a Wenger double pass Dryer/Cooler (Series 4800, Wenger Manufacturing Inc., Sabetha, KS, USA) at 104 °C for 10 min.

The dried extrudates were ground using a Schutte Buffalo Hammer mill (Buffalo, NY, USA). The ground binary blends were then mixed with sugar (Domino Foods, Inc., Yonkers, NY, USA), whey protein concentrate WPC80 (Davisco Foods International, Inc., Eden Prarie, MN, USA), antioxidant (BHA, butylated hydroxyanisole and BHT, butylated hydroxytoluene), vitamins and minerals (Research Products Company, Salina, KS, USA), and non-GMO soybean oil (Zeeland Farm Services, Inc., Zeeland, MI, USA). The composition of all blends is shown in Table 2.

#### 2.1.2. Current Non-Extruded Fortified Blended Food

Corn soy blend plus (CSB+) was produced by Bunge Milling (St. Louis, MO, USA) according to the USDA commodity requirements [19] (Table 2). 

### 2.2. Sample Preparation

All products were prepared into porridges, which are the most common dishes prepared from cereal-based commodities for children in developing countries [20,21,22], with 20% solids content according to the recommendation from [6].

A weighted FBF flour (200 g) was mixed with cold water (400 mL) to prevent the formation of lumps. The mixture was then added to boiling water (400 mL), brought back to a boil, cooked with continuous stirring with a wooden spoon for 2 min for extruded FBFs and 10 min for non-extruded FBFs. The sample was removed from the stovetop and cooled to a temperature of 45 °C, which is the typical consumption temperature by infant and young children [23].

### 2.3. Descriptive Sensory Analysis

Descriptive sensory analysis was conducted by six highly-trained panelists at the Center for Sensory Analysis and Consumer Behavior, Manhattan, Kansas USA. All of these panelists had completed 120 h of general descriptive analysis panel training, and had over 2000 h of evaluation experience with a wide array of food products, including cereal-based products.

Sixteen sensory attributes, including 6 aroma and 10 flavor, were evaluated in all samples (Table 3). Some of the same attributes were used in Chanadang et al. [23].

Fifty grams of each prepared porridge was served in a 4 oz styrofoam cup (Dart container corporation, Mason, MI, USA) and labeled with a three-digit code for each panelist. All samples were evaluated on a numerical scale of 0–15 with 0.5 increments, where 0 represents none and 15 represents extremely high. The samples were prepared and evaluated in triplicate in a randomized order.

### 2.4. Data Analysis

Sixteen sensory attributes were evaluated for all porridge samples, however, panelists did not detect rancid or painty characteristics in any samples. Therefore, twelve sensory attributes, besides rancid and painty characteristics, were reported and analyzed in this study.

Data for each sensory attribute was analyzed by a one-way ANOVA mixed effect model (SAS version 9.4, The SAS Institute Inc., Cary, NC, USA) using PROC GLIMMIX to determine significant differences (*p* ≤ 0.05) among porridge samples. Tukey’s HSD test was used at the 5% level of significance to locate significant effects of the sample on each sensory property. Principal component analysis (PCA) was performed in order to visualize the relationship among sensory attributes and samples using Unscrambler^®^ X 10.5 (Camo, Magnolia, TX, USA).

## 3. Results and Discussion

The results showed that six out of twelve sensory attributes were significantly different among porridge samples (*p* ≤ 0.05), including toasted and beany aroma and flavor, sweetness, and bitterness (Table 4).

Porridges prepared from novel extruded FBFs appeared to be higher in toasted aroma and flavor than non-extruded FBF (CSB+), although, not all novel extruded FBFs were significantly different from CSB+ in this sensory characteristic (*p* > 0.05). The high temperature used in the extrusion process might be the main reason for the increased toasted characteristic in extruded FBFs. Extrusion cooking of cereal normally involves thermally induced reactions, including the Maillard reaction, that could generate chemical compounds that correspond to a desirable aroma and flavor of the products [24,25], including such aspects as “toasted” sensory properties. Parker et al. [26] reported that extruded cereal samples with high levels of Maillard reaction products, such as pyrazines and sulfur-containing alicyclic compounds, were generally described as having a desirable toasted or roasted cereal aroma and flavor. Besides the extrusion process, other processing parameters, including types of milling, decortication process, and the step of adding antioxidant to the blends, did not show significant effects on sensory properties of FBFs in this study.

The composition of FBFs seemed to be another important factor that affected the sensory properties of the products. Porridges prepared from sorghum-cowpea blends, especially WSCB-V3, had significantly higher intensity in beany aroma and flavor (*p* ≤ 0.05) than the ones prepared from sorghum-soy and corn-soy blends. Beany characteristics are often found in legume-containing products and are attributed to the action of the lipoxygenase enzyme, which catalyzes the lipid oxidation of linolenic and linoleic fatty acids [27,28]. Since all of the products in this study contained legumes (either soybeans or cowpea), the difference in intensity in beany characteristics among products was primarily due to the amount of legume used in each blend. This probably explains why sorghum-cowpea blends with higher amounts of legume (38.6% cowpea) were higher in beany aroma and flavor.

The variety of sorghum used in FBFs might be another factor that affected the beany property of the products. The blend containing whole red sorghum flour (WSCB-V3) was significantly higher in beany flavor than the rest of the FBFs, except for the one that contained decorticated red sorghum flour (SCB-V3). Vara-Ubol et al. [29] indicated that beany was considered as a combination of attributes, including musty/dusty, musty/earthy, sour aromatics, and characterizing attributes such as green/pea pod, nutty or brown. Red sorghum varieties were reported to have higher dusty flavor [30] and porridges made with red sorghum were also reported to have higher overall flavor intensity [31]. FBFs with red sorghum variety in this study might be higher in dusty flavor or overall intensity, and that resulted in an increased intensity of beany characteristics. 

Porridges prepared from various FBFs were also significantly different in sweetness (*p* ≤ 0.05). As expected, novel extruded FBFs with the addition of 15% sugar were significantly higher in sweetness than the traditional non-extruded FBF (CSB+) (*p* ≤ 0.05). The addition of sugar into the FBFs formulation was not only to provide energy, but could also to increase the palatability and consumption of the products [6]. Iuel-Brockdorf et al. [32] also found that products with a sweeter flavor received better ratings in terms of child and caregiver acceptability.

Salt was significantly different among the FBFs porridges (*p* ≤ 0.05), however, it was only a small difference (lower than 0.5 points on a 15 point scale). The higher intensity of salt in novel extruded FBFs was probably due to the higher amount of vitamin and mineral premix that had been added into the formulation. Gilbertson et al. [33] indicated that the taste system plays important roles in nutrient identification and salty taste reflects the recognition of minerals in foods. The study by Teillet et al. [34] also found that a more salty taste was found in waters with higher mineral contents.

Porridge prepared from binary blends with higher levels of lipids, e.g., whole corn with full-fat soybean blend (WCS”B), was significantly higher in bitterness than most of the FBFs porridges (*p* ≤ 0.05). The high temperature used in the extrusion process could have accelerated the degradation of lipids, and the degraded lipids appeared to be associated with unpleasant flavors, such as astringent, bitter, and rancid [24,35,36]. WCS”B, which had high levels of lipid, was more likely to have a higher amount of degraded lipid after the extrusion process, and this could result in the higher bitter taste of the cooked porridge.

**Figure 1:**
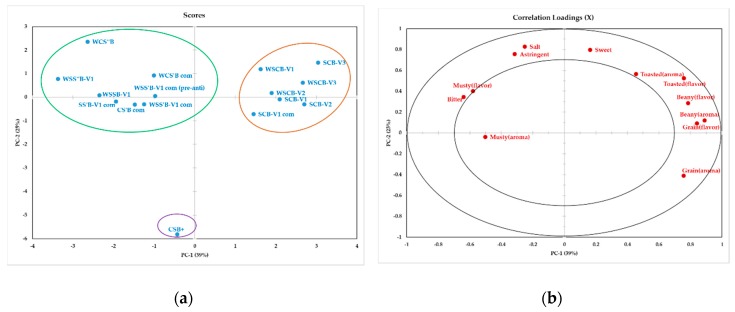
Principal component analysis of the porridges prepared from FBFs and sensory attributes (**a**) Score plot. (**b**) Correlation loading plot. For the FBFs, W = Whole, first S = Sorghum flour, first C = Degermed corn flour, second S = Low-fat soy flour, S’ = Medium-fat soy flour, S” = Full-fat soy flour, second C = Cowpea flour, V1 and V2 = White variety of sorghum, V3 = Red variety of sorghum, com = Commercial milling, (pre-anti) = Antioxidant had been added to the binary blend before extrusion process. CSB+ represents the control sample (current non-extruded FBF).

Principal component analysis (PCA) of twelve sensory attributes helped to visualize the differences among porridge samples (Figure 1). PC1 accounted for 39% of the variation, and seemed to differentiate among samples according to beany, toasted, grain, musty, and bitter attributes. PC2 accounted for 25% of the variation, and seemed to differentiate among samples according to flavor attributes, including astringency, sweetness, and saltiness. Current non-extruded FBF (CSB+) was separated from novel extruded FBFs due to the lower intensity in sweetness, saltiness, and astringency. Extruded corn-soy blends and extruded sorghum-soy blends were grouped together and had more pronounced bitter and musty attributes. As previously mentioned, the extruded products containing higher amount of lipids were more bitter (*p* ≤ 0.05) because of the high possibility of having more degraded lipids. However, it must be noted that the lipids certainly were not degraded enough to produce marked changes in shelf-life [17]. Phenolic compounds, which can be found in sorghum, are responsible for the bitterness of many similar foods and may cause a negative effect on products’ acceptability [35,37]. Therefore, the higher amount of sorghum (47.6% sorghum) used in sorghum-soy blend formulations was another reason that made those blends higher in bitter taste. This effect also was found in 20% solids FBFs made of sorghum without added sugar [38].

All extruded sorghum-cowpea blends were grouped together. They were mainly characterized by toasted, grain, and beany attributes. The sorghum-cowpea binary blend that was used to make extruded sorghum-cowpea blends had lower levels of lipids compared to sorghum-soy and corn-soy binary blends [39]. Feng and Lee [40] reported that during extrusion, the lipid worked as a lubricant, and decreased the temperature in the extruder barrel. The lower amount of lipids in the sorghum-cowpea blend contributed to higher friction between the particles in the mix and the screw surface, and directly related to a higher temperature in the extruder barrel. The higher temperature during the extrusion process could probably generate higher levels of chemical compounds from the Maillard reaction, which were responsible for desirable attributes, such as cereal-like, toasted, or roasted aromas [24,26].

## 4. Conclusions

The results from this study clearly identified the effects of the extrusion process and ingredients used on the sensory properties of the products. Novel FBFs from the extrusion process had more pronounced toasted characteristics due to the higher temperature during extrusion. The type of milling, decortication process, and the step of adding antioxidant to the blends did not show effects on the sensory properties of FBFs in this study. Adding sugar and increasing the amount of vitamin-mineral premix in the novel FBFs formulation increased the sweetness and saltiness of the products, respectively, as expected, which is not surprising given that caregivers have been shown to add sugar to current unsweetended FBFs. The level of lipids in binary blends was mainly responsible for the bitterness of the product. In addition, legumes, such as soybeans and cowpeas, were the main ingredient that contributed to the beany characteristics of the products. The higher amount of legume used in the formulations, the more beany characteristics that could be perceived from the products.

## Figures and Tables

**Table 1:** List of processing and ingredients used for each extruded fortified blended food (FBF).

Treatment		Product Code ^1^	Cereal			Legume
			Cereal Type	Variety	Milling Type	
1	Sorghum-Cowpea blend	SCB-V1 com	Sorghum-Decorticated	White-Fontanelle 4525	Commercial	Cowpea
2		SCB-V1	Sorghum-Decorticated	White-Fontanelle 4525	Pilot	Cowpea
3		SCB-V2	Sorghum-Decorticated	White-738Y	Pilot	Cowpea
4		SCB-V3	Sorghum-Decorticated	Red-217X Burgundy	Pilot	Cowpea
5		WSCB-V1	Sorghum-Whole	White-Fontanelle 4525	Pilot	Cowpea
6		WSCB-V2	Sorghum-Whole	White-738Y	Pilot	Cowpea
7		WSCB-V3	Sorghum-Whole	Red-217X Burgundy	Pilot	Cowpea
8	Sorghum-Soy blend	SS’B-V1 com	Sorghum-Decorticated	White-Fontanelle 4525	Commercial	Soybean—High Fat
9		WSSB-V1	Sorghum-Whole	White-Fontanelle 4525	Pilot	Soybean—Low Fat
10		WSS’B-V1 com	Sorghum-Whole	White-Fontanelle 4525	Commercial	Soybean—High Fat
11		WSS’B-V1 com (pre-anti)	Sorghum-Whole	White-Fontanelle 4525	Commercial	Soybean—High Fat
12		WSS’’B-V1	Sorghum-Whole	White-Fontanelle 4525	Pilot	Soybean—Full Fat
13	Corn-Soy blend	CS’B com	Corn-Degermed		Commercial	Soybean—High Fat
14		WCS’B com	Corn-Whole		Commercial	Soybean—High Fat
15		WCS’’B	Corn-Whole		Pilot	Soybean—Full Fat

^1^ W = Whole, first S = Sorghum flour, first C = Degermed corn flour, second S = Low-fat soy flour, S’ = Medium-fat soy flour, S” = Full-fat soy flour, second C = Cowpea flour, V1 and V2 = White variety of sorghum, V3 = Red variety of sorghum, com = Commercial milling, (pre-anti) = Antioxidant had been added to the binary blend before extrusion process.

**Table 2:** Composition of extruded FBFs and non-extruded FBFs.

Ingredients (%)	Extruded FBFs ^1^			Non-Extruded FBF
	Sorghum-Cowpea Blends (SCB)	Sorghum-Soy Blends (SSB)	Corn-Soy Blends (CSB)	Corn Soy Blend Plus (CSB+)
Sorghum flour	24.7	47.6		
Cowpea flour	38.6			
Corn flour			48.1	
Corn (White or Yellow)				78.5
Whole soybeans				20.0
Soy flour		15.7	15.2	
Sugar	15.0	15.0	15.0	
Whey Protein Concentrate (WPC80)	9.5	9.5	9.5	
Soybean oil	9.0	9.0	9.0	
Vitamin & Mineral Premix	3.1	3.1	3.1	
Antioxidant ^2^	0.1	0.1	0.1	
Vitamin/Mineral				0.2
Tri-Calcium Phosphate				1.2
Potassium Chloride				0.2

^1^ For extruded FBFs with full-fat soy, WPC80 was increased from 9.5 to 13.0%, and soybean oil was decreased from 9 to 5.5%. ^2^ Antioxidant was a mixture of 50% butylated hydroxyanisole (BHA) and 50% butylated hydroxytoluene (BHT).

**Table 3:** Aroma and flavor attributes, definitions, and references for descriptive analysis of porridge prepared from FBFs.

Attribute	Definition	Reference ^$^
*Aroma*		
Overall Grain *	A general term used to describe the aromatics which includes musty, dusty, slightly brown, slightly sweet and is associated with harvested grains and dry grain stems.	Cereal Mix(dry) = 7.5. Preparation: Mix ½ cup of each General Mills Rice Chex, Wheaties and Quaker Quick Oats. Put in a blender and “pulse” blend into small particles. Serve 2 Tablespoon in a 12 oz brandy snifter, covered with a watch glass.
Toasted *	A moderately browned/baked impression.	Crushed Post Shredded wheat = 2.5. Preparation: Crush ¼ cup of Shredded wheat and served in a 12 oz brandy snifter, covered with a watch glass.
		Crushed General Mills Cheerios = 7.0. Preparation: Crush ¼ cup of Cheerios and serve in a 12 oz brandy snifter, covered with a watch glass.
Beany	Aromatic characteristic of beans and bean products, includes musty/earthy, musty/dusty, sour aromatics, bitter aromatics, starchy and green/pea pod, nutty or brown.	Cooked Soy Bean = 4.0. Preparation: Soak ½ cup of soy bean overnight and boil the bean 2.5 h. Serve 1 table spoon of cooked soy bean in a 12 oz brandy snifter, covered with a watch glass.
		Bush Pinto Beans (canned) = 7.0. Preparation: Drain beans and rinse with de-ionized water Place one table spoon in a 12 oz brandy snifter, covered with a watch glass.
Musty Overall *	A combination of one or more aromatic impressions characterized to some degree as being somewhat dry, dusty, damp, earthy, stale, sour, or moldy. If identifiable, attribute will be listed.	1,2,4Trimethoxybenzene 50,000 ppm = 4.0. Preparation: Dip an Orlandi Perfumer Strip #27995 2.2 cm (second marking line) into solution and place dipped end up in a Fisherbrand Disposable Borosilicate Glass Tubes with Threaded End (15 × 150 mm) cap.
Rancid	A somewhat heavy aromatic characteristic of old, oxidized, decomposing fat and oil. The aromatics may include painty, varnish, or fishy.	Microwaved Wesson vegetable oil (4 min at high) = 2.5. Preparation: Microwave 1 ½ cups oil on high power for 4 min. Let cool and serve ¼ cup in a 12 oz brandy snifter covered with a watch glass.
		Microwaved Wesson vegetable oil (5 min at high) = 5.0. Preparation: Microwave 1 ½ cups oil on high power for 5 min. Let cool and serve ¼ cup in a 12 oz brandy snifter covered with a watch glass.
Painty	The aromatic associated with rancid oil and fat, typically in the late stages of rancidity.	Microwaved Wesson vegetable oil (4 min at high) = 2.5. Preparation: Microwave 1 ½ cups oil on high power for 4 min. Let cool and pour into 1 oz cups. Serve covered.
		Microwaved Wesson vegetable oil (5 min at high) = 4.5. Preparation: Microwave 1 ½ cups oil on high power for 5 min. Let cool and pour into 1 oz cups.
*Flavor*		
Overall Grain *	A general term used to describe the light dusty/musty aromatics associated with grains such as corn, wheat, bran, rice, oats and soybean.	Cereal Mix (dry) = 8.0. Preparation: Mix ½ cup of each General Mills Rice Chex, Wheaties and Quaker Quick Oats. Put in a blender and “pulse” blend into small particles. Serve in 1 oz cup.
Toasted *	A moderately browned/baked impression.	Post Shredded Wheat (Spoon size) = 3.5. Preparation: Serve in 3.25 oz cup.General Mills Cheerios = 7.0. Preparation: Serve in 3.25 oz cup.
Beany	Aromatic characteristic of beans and bean products, includes musty/earthy, musty/dusty, sour aromatics, bitter aromatics, starchy and green/pea pod, nutty or brown.	Cooked Soy Bean = 4.0. Preparation: Soak ½ cup of soy bean overnight and boil the bean 2.5 h. Serve in 1 oz cup.
		Bush Pinto Beans (canned) = 7.5. Preparation: Drain beans and rinse with de-ionized water Serve in 1 oz cup.
Musty *	Aromatics associated with wet grain and damp earth.	Cooked American Beauty elbow macaroni = 5.0. Preparation: Bring 3 cups water to a rapid boil. Add 1 cup pasta and stir, returning to a rapid boil. Cook 6 min, stirring occasionally. Drain and place into 3.25 oz cups.
Rancid	A somewhat heavy aromatic characteristic of old, oxidized, decomposing fat and oil. The aromatics may include painty, varnish, or fishy.	Microwaved Wesson vegetable oil (4 min at high) = 3.0. Preparation: Microwave 1 ½ cups oil on high power for 4 min. Let cool and serve in 1 oz cup.
		Microwaved Wesson vegetable oil (5 min at high) = 5.0. Preparation: Microwave 1 ½ cups oil on high power for 5 min. Let cool and serve in 1 oz cup.
Painty	The aromatic associated with rancid oil and fat, typically in the late stages of rancidity.	Microwaved Wesson vegetable oil (4 min at high) = 0.0. Preparation: Microwave 1 ½ cups oil on high power for 4 min. Let cool and serve in 1 oz cup.
		Microwaved Wesson vegetable oil (5 min at high) = 3.0. Preparation: Microwave 1 ½ cups oil on high power for 5 min. Let cool and serve in 1 oz cup.
Sweet *	A fundamental taste factor of which sucrose is typical.	2% Sucrose Solution = 2.0
		4% Sucrose Solution = 4.0
Salt *	Fundamental taste factor of which sodium chloride is typical.	0.15% Sodium Chloride Solution = 1.5
		0.20% Sodium Chloride Solution = 2.5
Bitter *	The fundamental taste factor associated with a caffeine solution.	0.01% Caffeine Solution = 2.0
		0.02% Caffeine Solution = 3.5
		0.035% Caffeine Solution = 5.0
		0.05% Caffeine Solution = 6.5
Astringent *	The drying, puckering sensation on the tongue and other mouth surfaces.	0.050% alum solution = 2.5
		0.100% alum solution = 5.0

^$^ 0 to 15 point numeric scale with 0.5 increments was used to rate the intensities of the sample and references. * From Chanadang and others (2016).

**Table 4:** Mean scores ^1^ (standard error) of sensory attributes for porridges prepared from FBFs.

Treatment ^2^	Overall Grain (a) ^3^	Toasted (a)	Beany (a)	Musty Overall (a)	Overall Grain (f)	Toasted (f)	Beany (f)	Musty (f)	Sweet (f)	Salt (f)	Astringent (f)	Bitter (f)
**SCB-V1 com**	7.14 (0.07)	3.53 ^ab4^ (0.18)	3.28 ^abc^ (0.19)	3.36 (0.16)	7.36 (0.07)	2.97 ^abc^ (0.20)	3.58 ^bcd^ (0.15)	4.47 (0.15)	2.11 ^a^ (0.16)	1.42 ^ab^ (0.15)	2.64 (0.17)	2.89 ^d^ (0.18)
**SCB-V1**	7.17 (0.07)	3.89 ^ab^ (0.29)	3.28 ^abc^ (0.24)	3.11 (0.21)	7.36 (0.10)	3.28 ^abc^ (0.27)	3.64 ^bc^ (0.24)	4.36 (0.22)	2.03 ^a^ (0.15)	1.39 ^ab^ (0.15)	2.81 (0.25)	3.17 ^bcd^ (0.18)
**SCB-V2**	7.25 (0.08)	4.47 ^a^ (0.23)	3.19 ^abc^ (0.13)	3.17 (0.18)	7.42 (0.09)	3.22^abc^ (0.16)	3.64^bc^ (0.16)	4.33 (0.21)	2.00 ^a^ (0.19)	1.31 ^ab^ (0.14)	2.81 (0.15)	3.08 ^cd^ (0.20)
**SCB-V3**	7.22 (0.07)	4.53 ^a^ (0.30)	3.36 ^ab^ (0.19)	2.94 (0.19)	7.36 (0.09)	3.75 ^a^ (0.31)	4.19 ^ab^ (0.14)	4.69 (0.22)	1.97 ^a^ (0.12)	1.58 ^a^ (0.20)	2.68 (0.19)	3.31 ^bcd^ (0.13)
**WSCB-V1**	7.11 (0.08)	4.28 ^ab^ (0.18)	3.25 ^abc^ (0.19)	3.19 (0.18)	7.39 (0.08)	3.50 ^ab^ (0.23)	3.50 ^bcde^ (0.17)	4.58 (0.23)	2.00 ^a^ (0.10)	1.58^a^ (0.15)	2.78 (0.18)	3.28 ^bcd^ (0.20)
**WSCB-V2**	7.22 (0.07)	3.83 ^ab^ (0.24)	3.14 ^abc^ (0.18)	3.06 (0.18)	7.44 (0.08)	3.11 ^abc^ (0.23)	3.64 ^bc^ (0.18)	4.75 (0.22)	2.03 ^a^ (0.12)	1.50 ^ab^ (0.16)	2.72 (0.19)	2.97 ^d^ (0.19)
**WSCB-V3**	7.19 (0.07)	3.67 ^ab^ (0.23)	3.89 ^a^ (0.17)	3.44 (0.21)	7.33 (0.08)	3.33 ^abc^ (0.27)	4.44 ^a^ (0.19)	4.39 (0.24)	2.06 ^a^ (0.14)	1.47 ^ab^ (0.20)	2.83 (0.23)	3.36 ^bcd^ (0.20)
**SS’B-V1 com**	6.92 (0.10)	3.56 ^ab^ (0.21)	2.72 ^bc^ (0.21)	3.47 (0.22)	7.17 (0.07)	2.75 ^abc^ (0.11)	3.19 ^cde^ (0.10)	4.75 (0.18)	1.89 ^a^ (0.14)	1.31 ^ab^ (0.13)	2.97 (0.19)	3.31 ^bcd^ (0.17)
**WSSB-V1**	6.92 (0.06)	2.97 ^b^ (0.14)	2.69 ^bc^ (0.21)	3.19 (0.20)	7.19 (0.08)	2.36 ^c^ (0.13)	3.39 ^cde^ (0.21)	4.94 (0.25)	1.97^a^ (0.17)	1.58 ^a^ (0.18)	2.75 (0.13)	3.47 ^bcd^ (0.14)
**WSS’B-V1 com**	7.03 (0.16)	3.72 ^ab^ (0.21)	2.61 ^bc^ (0.16)	3.22 (0.18)	7.14 (0.17)	2.69 ^bc^ (0.14)	3.11 ^cde^ (0.15)	4.69 (0.21)	2.17 ^a^ (0.18)	1.44 ^ab^ (0.18)	2.67 (0.17)	3.22 ^bcd^ (0.18)
**WSS’B-V1 com (pre-anti)**	7.06 (0.08)	3.58 ^ab^ (0.19)	2.75 ^bc^ (0.18)	3.36 (0.13)	7.19 (0.06)	3.00 ^abc^ (0.16)	3.28 ^cde^ (0.16)	4.72 (0.18)	1.94 ^a^ (0.15)	1.44 ^ab^ (0.15)	2.86 (0.18)	3.31 ^bcd^ (0.21)
**WSS’’B-V1**	7.00 (0.07)	3.00 ^b^ (0.17)	2.56 ^c^ (0.18)	3.75 (0.20)	7.25 (0.09)	2.50 ^bc^ (0.16)	3.17 ^cde^ (0.18)	4.94 (0.21)	1.86 ^a^ (0.08)	1.64 ^a^ (0.18)	3.06 (0.19)	3.81 ^abc^ (0.19)
**CS’B com**	6.94 (0.09)	3.89 ^ab^ (0.22)	2.64 ^bc^ (0.11)	3.28 (0.18)	7.19 (0.12)	2.53 ^bc^ (0.14)	3.03^cde^ (0.14)	4.42 (0.27)	2.03 ^a^ (0.17)	1.47 ^ab^ (0.17)	2.72 (0.23)	3.58 ^bcd^ (0.17)
**WCS’B com**	7.08 (0.14)	4.22 ^ab^ (0.24)	2.58 ^bc^ (0.16)	3.22 (0.19)	7.17 (0.11)	3.19 ^abc^ (0.19)	2.89 ^de^ (0.10)	4.50 (0.19)	2.11 ^a^ (0.19)	1.67 ^a^ (0.19)	2.67 (0.22)	3.89 ^ab^ (0.20)
**WCS’’B**	7.03 (0.10)	4.50 ^a^ (0.24)	2.64 ^bc^ (0.18)	3.17 (0.14)	7.06 (0.07)	3.03 ^abc^ (0.14)	2.89 ^de^ (0.17)	4.75 (0.23)	1.89 ^a^ (0.21)	1.69 ^a^ (0.21)	3.08 (0.18)	4.53 ^a^ (0.20)
**CSB+**	7.33 (0.11)	2.97 ^b^ (0.20)	2.75 ^bc^ (0.19)	3.22 (0.15)	7.17 (0.11)	2.36 ^c^ (0.10)	2.83 ^e^ (0.15)	4.36 (0.18)	0.86 ^b^ (0.13)	1.14 ^b^ (0.13)	2.28 (0.14)	3.39 ^cde^ (0.18)

^1^ Scores are based on a 0–15-point numeric scale with 0.5 increments (0 = none and 15 = extremely high). Each mean score intensity was calculated from six panelists with three replicates. ^2^ W = Whole, first S = Sorghum flour, first C = Degermed corn flour, second S = Low-fat soy flour, S’ = Medium-fat soy flour, S” = Full-fat soy flour, second C = Cowpea flour, V1 and V2 = White variety of sorghum, V3 = Red variety of sorghum, com = Commercial milling, (pre-anti) = Antioxidant had been added to the binary blend before extrusion process. ^3^ (a) = Aroma, (f) = Flavor ^4^_._ Average for each parameter with a different letter in the same column were significantly different (*p* ≤ 0.05) between treatments.
